# A minimally invasive treatment option for large metastatic brain tumors: long-term results of two-session Gamma Knife stereotactic radiosurgery

**DOI:** 10.1186/1748-717X-9-132

**Published:** 2014-06-10

**Authors:** Shoji Yomo, Motohiro Hayashi

**Affiliations:** 1 Division of Radiation Oncology, Aizawa Comprehensive Cancer Center, Aizawa Hospital, Matsumoto, Japan; 2 Saitama Gamma Knife Center, San-ai Hospital, Saitama, Japan

**Keywords:** Brain metastases, Stereotactic radiosurgery, Gamma knife

## Abstract

**Background:**

Large brain metastases (BM) remain a significant cause of morbidity and death for cancer patients despite current advances in multimodality therapies. The goal of the present study was to evaluate the efficacy and limitations of 2-session Gamma Knife stereotactic radiosurgery (SRS) for patients with large BM.

**Methods:**

This is a prospective, open-label and single arm study analyzing 58 consecutive patients who received 2-session SRS for large BM (≥ 10 mL). The median age was 66 years, and the median Karnofsky performance status (KPS) score was 70. SRS was the initial treatment in 51 large tumors (84%) and was used as salvage after failed prior treatments for 10 tumors (16%). The fraction protocol was 20-30 Gy given in 2 fractions with 3–4 weeks between fractions. Overall survival (OS) and neurological death (ND), local tumor control and KPS were analyzed.

**Results:**

The median follow-up time was 9.0 months. One- and 2-year OS rates were 47% and 20%, respectively. The median OS time was 11.8 months (95% CI: 5.5-15.6). The causes of death were intracranial local progression in 5 cases, meningeal carcinomatosis in 3 and progression of the primary lesion in 39. One- and 2-year ND-free survival rates were 91% and 84%, respectively. In 52 of 61 large BM (85%) with sufficient radiological follow-up data, 6- and 12-month local tumor control rates were 85% and 64%, respectively. The mean KPS improved from 70 at the 1st SRS to 82 at the 2nd; the first follow-up mean KPS was 87 (*P* < 0.001). Symptomatic radiation injury developed and required conservative treatment in 3 patients (5%).

**Conclusions:**

Long-term follow-up showed that two-session Gamma Knife SRS achieved durable tumor control rates as well as acceptable treatment-related morbidity. This treatment method may potentially merit being offered to patients with large BM who are in poor condition or are otherwise ineligible for standard care.

## Background

Large brain metastases (BM) (≥ 10 mL) present a therapeutic dilemma, as the dose delivered by stereotactic radiosurgery (SRS) in a single fraction is limited by toxicity to adjacent tissues, resulting in suboptimal local control [1,2]. Hypofractionated stereotactic radiotherapy (SRT) is increasingly being applied to improve the therapeutic ratio between the probabilities of tumor control and normal tissue complications [3-7]. On the other hand, hypofractionated SRT was recently reported to be less effective than single-dose SRS in radioresistant tumor subtypes [8]. The clinical benefit of dose fractionation is still regarded as being undetermined. The authors previously reported the preliminary results of an alternative treatment protocol consisting of 2-session stereotactic radiosurgery (SRS) for large metastatic brain tumors (volume > 15 mL in the supratentorial region or > 10 mL in the infratentorial region), which suggested a priori treatment to be a safe modality providing neurological palliation in the short- to medium-term, with acceptable tumor control rates and low morbidity [9]. In line with the results of our pilot study, we continued to accumulate experience with this treatment strategy in order to elucidate its long-term efficacy and safety. Prognostic factors related to patient survival and local tumor control rates were also investigated.

## Methods

### Patient population

Based on previous results obtained in our pilot study, the inclusion criteria were extended as follows: i) patients with large BM (volume ≥ 10 mL regardless of prior treatment), ii) tumors not causing clinical signs of impending cerebral herniation, iii) tumors ineligible for surgical resection due to inaccessibility, the number of intracranial lesions and systemic disease states. In principle, surgical resection was recommended for large BM causing neurological symptoms refractory to corticosteroid therapy. In the event of surgery not being feasible, 2-session SRS was carefully conducted. All patients and/or their relatives were fully informed that 2-session SRS remains an unproven strategy in terms of safety and efficacy, and all provided written informed consent. San-ai Hospital Institutional Review Board approved this prospective clinical trial in September 2009.

Between September 2009 and July 2013, 60 consecutive patients with large BM were eligible for the present prospective clinical trial. However two of these patients could not complete the treatment protocol due to systemic disease progression. Thus the current study included a series of 58 patients with 61 large BM who completed 2-session SRS. Thirty-seven patients were male and 21 were female with a median age of 66 years (range: 32–88 years). The median Karnofsky performance status (KPS) score at the time of SRS was 70 (range: 30–100) and there were 3, 27 and 28 patients, respectively, in recursive partitioning analysis (RPA) classes I, II and III [10]. The median interval between primary diagnosis and initial SRS was 12.2 months (range: 0–192 months). In 17 patients (29%), neurological deficits caused by large BM were the initial symptoms of cancer. Forty-five patients (78%) had active systemic disease and/or extra-central nervous system (CNS) metastases and 21 (36%) underwent systemic chemotherapy at approximately the time of the initial SRS. Twenty-six patients (45%) had a single BM, the others multiple BM. The median number of BM at the initial SRS was 2 (range: 1–8). Two-session SRS was conducted as an initial treatment for 51 tumors (84%) and as a salvage procedure for 10 tumors (16%). Microsurgical resection for BM had been performed before SRS in 14 patients and prior whole brain radiotherapy (WBRT) had been conducted at the referring regional hospitals in 4 others. Patient characteristics are summarized in Table 1.

**Table 1:** Summary of clinical data from 58 consecutive patients with 61 large BM

**Characteristics**	**Values**
Sex (male/female)	37/21
Age (years), median (range)	66 (32–88)
KPS, median (range)	70 (30–100)
Active Extra-CNS disease/Extra-CNS metastasis	45 (76%)
RTOG-RPA class I/class II/class III	3/27/28
Primary cancer	
Lung	34
Breast	7
Colon & Rectus	7
Ovary	2
Double cancer	3
Other	5
No. of intracranial lesions, median (range)	2 (1–8)
Tumor location	
Supratentorial	41
Infratentorial	20
Procedures prior to SRS	
Craniotomy	14
Ommaya reservoir	10
WBRT	4

BM = brain metastases, KPS = Karnofsky performance status, CNS = central nervous system, RTOG-RPA = radiation treatment oncology group recursive partitioning analysis, SRS = stereotactic radiosurgery, WBRT = whole brain radiotherapy.

### Radiosurgical techniques

Gamma Knife SRS was performed using the Leksell G stereotactic frame (Elekta Instruments, Stockholm, Sweden). The frame was placed on the patient’s head under local anesthesia and with mild sedation. All patients underwent both stereotactic magnetic resonance (MR) imaging and computed tomography (CT). High-resolution 3-D volumetric gadolinium-enhanced T1-weighted images and 2 mm in thickness T2-weighted images were used for dose planning with Leksell Gamma Plan software (Elekta Instruments). The fraction protocol for large BM was consistent with that used in our previous study: 20-30 Gy in 2 fractions with 3–4 weeks between fractions. However, in some patients it was necessary to postpone the second procedure due to the schedule for systemic chemotherapy. The fractionated dose was calculated using a linear quadratic (LQ) formula, as described by Brenner et al. [11,12]. Assuming alpha/beta to be 10 for BM, 20–30 Gy in two fractions was approximately equivalent to a single administration of 16–23 Gy. The median tumor volume (TV) of large BM at the 1st session was 16.4 mL (range: 10.0-56.1) and the median dose prescribed was 14 Gy (range: 10–16) at the 45% isodose (range: 40–52). The median TV at the 2nd session was 8.9 mL (range: 2.3-42.6) and the median dose prescribed was 14 Gy (range: 10–15) at the 45% isodose (range: 40–60). The dosimetric profiles of 2-session SRS are summarized in Table 2. Synchronous small- to medium-sized metastases were treated with SRS at prescription doses ranging from 18 Gy to 22 Gy (median: 20 Gy) at either the first or the second session. The Leksell Gamma Knife Model C or Perfexion was used in all cases.

**Table 2:** Radiosurgical parameters

**Parameters**	**Median values (range)**	
	**1st SRS**	**2nd SRS**
Tumor volume (mL)	16.4 (10.0-56.1)	8.9 (2.3-42.6)
Prescribed isodose volume (mL)	18.4 (10.8-56.7)	11.7 (3.6-45.9)
Prescribed dose (Gy)	14 (10–16)	14 (10–15)
Prescribed isodose (%)	45 (40–52)	45 (40–60)
Maximum dose (Gy)	31.1 (20–38.1)	29.2 (22.2-35.7)
D95 (Gy)	14.7 (10.2-18.6)	15.0 (9.6-17.5)

SRS = stereotactic radiosurgery, D95 = the dose 95% of the target volume receives.

### Post-SRS management and follow-up evaluation

In most patients with neurological symptoms, administration of oral steroids (dexamethasone 2–4 mg/day) was maintained between the two sessions and then tapered off over a maximum of 4 weeks after the second session. Clinical follow-up data as well as contrast-enhanced MR images were obtained every one to three months. Local control failure was defined as an increase in target lesion diameter of at least 20% as compared to the smallest documented TV on MR images, irrespective of whether the lesion was a true recurrence or delayed radiation injury. Delayed radiation injury was differentiated from tumor recurrence using serial MR imaging and, in selected cases, ^11^C-methionine positron emission tomography [13]. Salvage SRS was possible provided that the volume of the local tumor recurrence was small enough for single-dose SRS. If metachronous remote metastases were documented, they were also principally managed with repeat SRS. Surgical removal was indicated when neurological signs became refractory to conservative management, with a radiological diagnosis of local tumor progression or radiation necrosis. When leptomeningeal carcinomatosis or miliary parenchymal metastases were documented, WBRT was then considered unless it had been used previously. Toxic effects were recorded and graded according to the National Cancer Institute Common Terminology Criteria for Adverse Events (CTCAE), version 3.0. Before closing the research database for analysis, the authors updated the follow-up data of patients who had not visited our outpatient department for more than two months. Inquiries about the date and mode of death were made by directly corresponding with the referring physician and/or the family of the deceased patient. Neurological death (ND) was defined as death attributable to CNS metastases including tumor recurrence and/or carcinomatous meningitis.

### Statistical analysis

The OS rate was calculated by the Kaplan-Meier product limit method. The ND rate was calculated employing Gray’s test [14], where death due to systemic disease progression was regarded as a competing event. For estimation of local tumor control rates, Gray’s test was used, with subsequent WBRT for other intracranial diseases being regarded as competing events. All of the above time-dependent analyses were based on the interval from the date of initial SRS treatment until the date of each event. To assess the impact on patient quality of life, KPS scores at each clinical stage were analyzed by the Friedman test. The Cox or Fine-Gray proportional hazards model [15] was employed, as appropriate, to investigate prognostic factors for OS and local tumor control rates. Prognostic candidates were selected with reference to previous studies [9,10,16,17]. A statistical processing software package, the “R” version 3.0.1 (The R Foundation for Statistical Computing, Vienna, Austria), was used for all statistical analyses. A *P*-value < 0.05 was considered to indicate a statistically significant difference.

## Results

**Figure 1:**
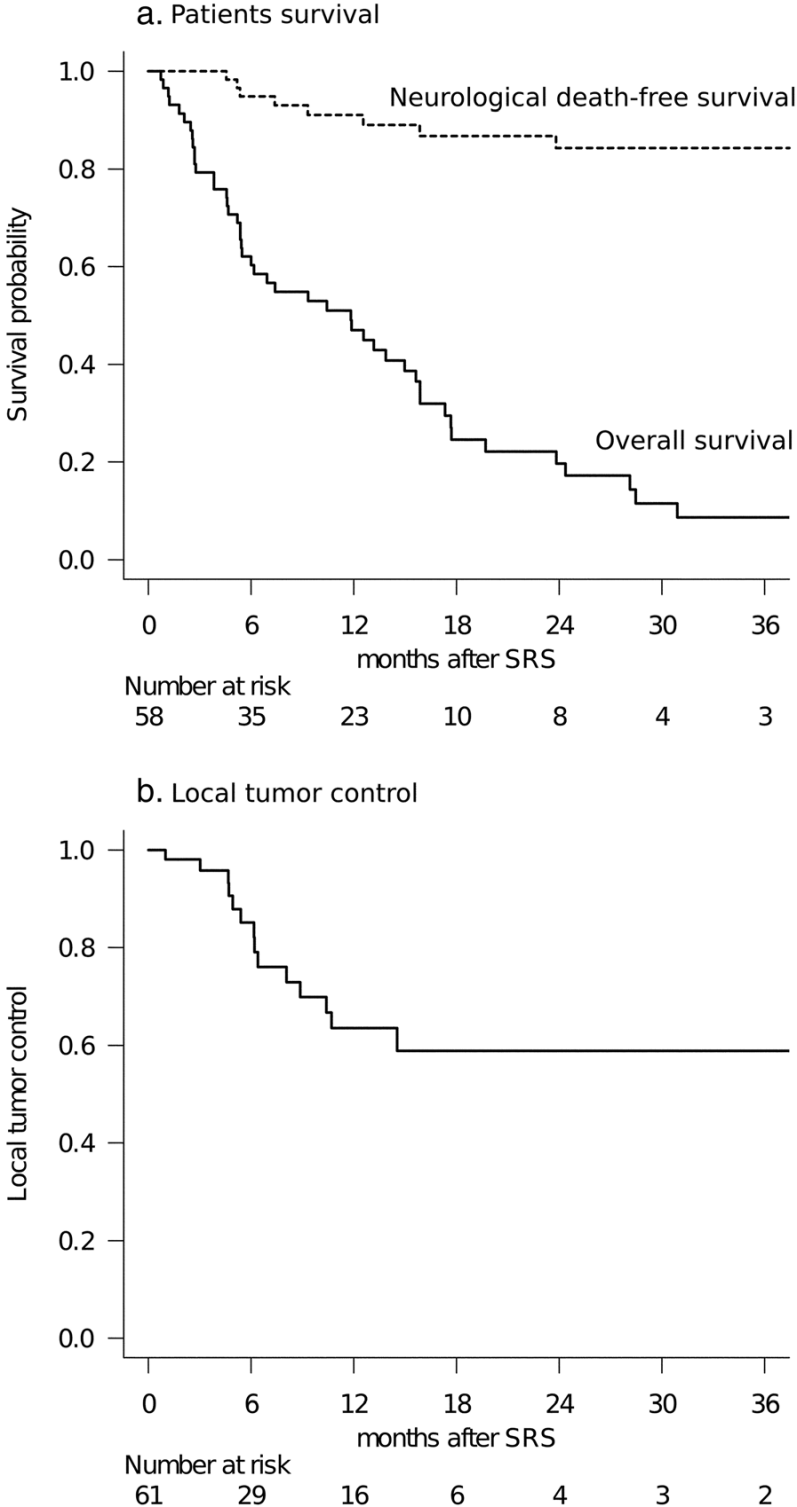
**Results of patient survival and local tumor control. (a)** Survival results: The solid line represents overall survival (OS) probability. The median survival time was 11.8 months (95% CI: 5.5-15.6). One- and 2-year OS rates after stereotactic radiosurgery (SRS) were 47% and 20%, respectively. The dotted line represents the neurological death (ND)-free survival probability adjusted for competing events. The 1- and 2-year ND-free survival rates after SRS were 91% and 84%, respectively. Note that the distance between these two lines represents the cumulative incidence of non-neurological death. **(b)** Local tumor control rates: Six- and 12-month local tumor control rates were 85% and 64%, respectively.

The median follow-up was 9.0 months and no patients were lost to follow-up. At the time of assessment, 11 patients (19%) were alive and 47 (81%) had died. One- and 2-year OS rates were 47% (95% CI: 34–59) and 20% (95% CI: 10–32), respectively (Figure 1a). The median OS time was 11.8 months (95% CI: 5.5-15.6). The proportional hazards model for OS is shown in Table 3. Controlled systemic disease (HR: 0.151, 95% CI: 0.057-0.405, *P* < 0.001), short interval from diagnosis to SRS (HR: 0.274, 95% CI: 0.127-0.593, *P* = 0.001) and a single BM (HR: 0.330, 95% CI: 0.166-0.656, *P* = 0.002) were identified as favorable prognostic factors independently predicting OS rates. The causes of death were local progression of large BM in 5 patients, meningeal carcinomatosis in 3 and progression of the primary lesion in 39. One- and 2-year ND-free survival rates were 91% (95% CI: 82–97) and 84% (95% CI: 73–93), respectively (Figure 1a).

**Table 3:** Analysis of factors predicting patient survival after 2-session SRS (Cox proportional hazards model)

**Covariate**	**Hazard ratio (95% CI)**	***P *** **value**
Young (≤ 65 y/o)	0.591 (0.299-1.17)	0.130
High KPS (≥ 90)	0.803 (0.437-1.48)	0.480
Controlled Extra-CNS disease	0.151 (0.057-0.405)	< .001
Short interval from cancer diagnosis to SRS (≤ 12 months)	0.274 (0.127-0.593)	0.001
Single BM	0.330 (0.169-0.656)	0.002

SRS = stereotactic radiosurgery, CI = confidence interval, KPS = Karnofsky performance status, CNS = central nervous system, BM = brain metastasis.

In total, 52 of the 61 large BM (85%) had sufficient radiological follow-up data, and local tumor control rates were evaluated using these data because 9 patients died from extra-CNS progression before the first MR imaging follow-up examination could be performed. Fourteen large BM were eventually diagnosed as local control failures at a median of 6.2 months (range: 1.0-14.5) after the initial session. Six- and 12-month local tumor control rates were 85% and 64%, respectively (Figure 1b). The proportional hazards model demonstrated TV decrease by more than half between two sessions to be the sole factor predicting higher local tumor control (HR: 0.087 95% CI: 0.009-0.832, *P* = 0.034) (Table 4). Salvage SRS was carefully applied for 8 large BM diagnosed as local recurrences. Although local control was achieved again in 6 cases, one of the remaining two continued to show tumor growth and the other developed the complication of delayed radiation injury, described in detail below. Microsurgery was required for three patients at a median of 4.9 months after the initial SRS (range: 1.0-14.7). Salvage WBRT was required in three patients at a median of 14.4 months after the initial SRS (range: 7.6-21.1) due to the subsequent development of multiple BM and/or leptomeningeal dissemination.

**Table 4:** **Analysis of factors predicting local tumor control after 2-session SRS (Fine-Gray proportional hazards model**)

**Covariate**	**Hazard ratio (95% CI)**	***P *** **value**
Prior local treatment	2.48 (0.489-12.5)	0.270
Large tumor volume (> 20 mL)	0.600 (0.119-3.01)	0.530
High cumulative dose (> 30 Gy as D95)	0.261 (0.068-1.01)	0.051
Significant tumor volume decrease at 2nd session	0.087 (0.009-0.832)	0.034

SRS = stereotactic radiosurgery, CI = confidence interval, D95 = the dose 95% of the target volume receives.

**Figure 2:**
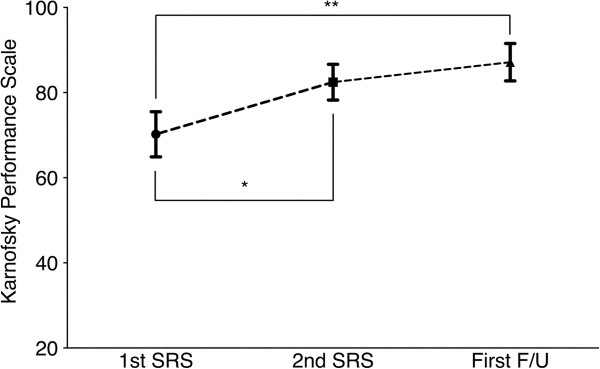
**Functional outcomes.** Graph showing the changes in Karnofsky performance status (KPS), with scores being 70 at the 1st stereotactic radiosurgery (SRS), 82 at 2nd SRS and 87 at the first follow-up visit (*P* < .001, Friedman test). There are significant trends toward improvement in patients’ performances after 2-session SRS (Bonferroni correction, *p < 0.001, **p < 0.001). Values are expressed as means ± 95% confidence interval.

In all 49 patients with post-SRS neuroimaging follow-up, neurological status was evaluated at each visit. The mean KPS improved significantly from 70 (95% CI: 65–75) at the 1st session to 82 (95% CI: 78–87) at 2nd; the first follow-up mean KPS score was 87 (95% CI: 83–92) (*P* < .001, Friedman test) (Figure 2). Eighteen of 22 patients whose pre-SRS KPS had decreased to less than 70 regained their independence in activities of daily living (KPS of 70 or more). On the other hand, four patients showed worsening of KPS at the first post-SRS evaluation. In two of these cases, deterioration was due to systemic disease progression while the others suffered persistent neurological symptoms.

As to adverse effects, there were no cases with NCI-CTCAE grade 4 toxicities in the current series. Two patients had transient emesis, and both required brief hospitalization for intravenous steroid administration (CTCAE grade 3 toxicity). Symptomatic delayed radiation injury developed and necessitated conservative treatment in three patients (CTCAE grade 3 toxicity), eventually showing clinical and radiological stabilization. Of these, a woman who received salvage SRS for local recurrence of large BM needed further repeat bevacizumab treatment for refractory radiation injury, but this management ultimately had to be discontinued because of progressive anemia probably related to the bevacizumab.

## Discussion

BM currently represent an important cause of cancer morbidity and mortality. The rationalized use of surgical resection, WBRT and SRS is key to successful treatment. Surgical resection has been a mainstay for large BM causing neurological deficits secondary to a mass effect, and this strategy can immediately eliminate neurological symptoms [18]. The combined approach of microsurgery followed by adjuvant radiotherapy has been recommended, if feasible, as means of decreasing both local and remote recurrence [19-22]. However, radical surgical resection of metastatic brain tumors in deep or eloquent locations may not necessarily be feasible because of the potential for neurological complications. Other factors such as a patient age, systemic disease progression and short life expectancy may make invasive treatment an unattractive option. SRS has been proved to be a safe and effective alternative for patients with small- to medium-sized BM. The delivery of highly focused radiation with a sharp dose fall-off is theoretically expected to reduce delayed neurotoxicity, and this feature makes it applicable even for patients in poor condition. However, tumor size strongly correlates with decreased responsiveness to radiation and an increased risk of neurotoxicity [23]. Dose fractionation was deemed to be a potential alternative strategy for increasing the total dose delivered to the lesion as well as controlling the toxicity to surrounding brain tissues. Although several studies have shown the efficacy of hypofractionated SRT for large BM [3-7], this treatment strategy without surgical excision has not yet been established for large BM. Recently Oermann et al. reported that hypofractionated SRT is less effective than single-dose SRS in radioresistant tumor subtypes [8]. The clinical benefit of dose fractionation for patients with relatively larger BM has yet to be determined.

The dose protocol for 2-session SRS was based on the LQ model, to allow adjustment for the difference between a single session and two sessions. There is an argument as to whether the LQ model is appropriate for large doses per fraction. The possibility of additional biological effects resulting from endothelial cell damage, enhanced tumor immunity, or both has been suggested [24,25]. However, we do not yet have an appropriate model taking into account these additional factors. Brown et al. recently reported that, for most tumors, the LQ model is still relevant for explaining the results obtained from clinical studies of SRS and SRT [12]. The clinical results obtained in the present study appear to support the validity of the LQ model in cases receiving a large dose per fraction. One of the most characteristic aspects of our treatment technique was the long inter-fraction time schedule, entirely different from that of standard hypofractionated SRT. Higuchi et al. reported 3-session SRS for large BM, wherein a total of 30 Gy was given in three fractions with a 2-week inter-fraction time, and their treatment results were quite impressive [4]. By referring to their treatment method, we developed our 2-session SRS protocol with a longer inter-fraction time. Based on extensive experience with single-dose SRS, at least two weeks are necessary for the resolution of patients’ symptoms after the intervention. Thus, we speculated that a three- to four-week inter-fraction time would be clinically meaningful. In fact, at the time of the second session, most patients came back with improved neurological conditions, mainly because of significantly reduced peritumoral edema, and most large BM were smaller than they had initially been. Longer inter-fraction time would allow a marked tumor size reduction, which, in turn, would enhance the safety of the second radiation treatment. Although clinical outcomes of the present study suggested that the radiobiological effect of a long inter-fraction time did not negatively influence local tumor control, from the viewpoint of radiation biology, a long inter-fraction time theoretically carries a potential risk, regarding kinetics, of tumor cell “repopulation” after the initial radiation. The effects of the repair of sublethal damage to DNA on the efficacy of treatment should be taken into account. On the other hand, it is also likely that hypoxic tumor cells become oxygenated during the inter-fraction time. Although the biological effect of irradiation must be a far more complex process, repopulation and reoxygenation may counterbalance each other even in the long inter-fraction setting [26].

Local control results for large BM after 2-session SRS were not better than those obtained in previously reported studies [4,7,27]. The definition of local control failure based on the Response Evaluation Criteria in Solid Tumors (RECIST) guidelines is stricter than that in other studies, where tumor size on follow-up gadolinium-enhanced MR images is compared with that at the time of treatment [4,17]. Such strict diagnostic criteria contributed to making this treatment strategy relevant, because salvage SRS could be applied in a timely manner when close image monitoring led to early detection of local recurrence. In fact, eight patients needed salvage SRS for local recurrence of large BM, and six of these were successfully managed. As the time-dependent curve of local tumor control indicated, local recurrences of large BM tended to be observed mostly within the first year after 2-session SRS and durable tumor control could be expected beyond this period. This means that vigilant follow-up in the first year after SRS is crucial for successful management of large BM. In the present series, a decrease in TV by more than half between the two sessions was the sole factor predicting a high local tumor control rate for large BM. This observation is consistent with the results recently reported by Sharpton et al. [28] and appears to be both clinically rational and relevant.

The results of applying local therapy to the brain are likely to be confounded by the competing high risk of death from extracranial disease progression. The authors believe it to be important to ascertain how SRS might prevent the patient from succumbing to intracranial disease, with adequate maintenance of quality of life. Thus, in the present study, we additionally conducted a competing risk analysis for appropriate evaluation of the efficacy of such local treatment and demonstrated that ND could be delayed or prevented in most patients by continuing radiosurgical management. Our experience suggests that the low incidence of ND might contribute to prolonging OS. Considering that patients with large BM usually have shorter survival durations [4,7,16,17,27], we believe the survivals in the present series to be long enough to merit offering this treatment strategy to patients with large BM who are in poor general condition.

With regard to prognostic factors that still need to be explained, the pre-SRS KPS score, already a validated prognostic factor in cancer patients [10,29], failed to predict patient survival. The KPS score decrease was mainly neurogenic in origin and was due to large BM in many patients. TV reduction following this treatment could provide neurological palliation, even for patients with low KPS scores (Figure 2). Once the local tumor causing neurological deficits is controlled, patients will have opportunities to be treated for their primary cancers.

Our SRS treatment strategy has not yet been validated and its safety is of major concern. The challenging problem of distinguishing local recurrence from radiation injury in surgically inaccessible areas remains. Radiological findings as well as clinical course should be taken into account in order to make an accurate diagnosis. However, the significance of changes in imaging findings remains uncertain. In fact, in two cases in which an accurate diagnosis could not be made despite exhaustive efforts, the only treatment option was supportive care. Furthermore, the appropriate management of delayed radiation injury is a matter of critical importance. In the present series, three patients required prolonged steroid administration and one of them further needed repeat bevacizumab treatment for prolonged symptomatic radiation injury [30,31].

The results of the present study must be interpreted with caution. As mentioned above, there is inherent selection bias because this was an open-label and single arm study. Thus the current study cannot address the potential role of SRS in comparison to surgical resection, the current standard of care for large BM. It is likely that patients with relatively mild neurological symptoms despite large intracranial tumors were assigned to 2-session SRS. In our opinion, the use of 2-session SRS should be limited to carefully selected patients who are ineligible for surgical resection and/or standard radiotherapy.

## Conclusion

The present results confirm those of our earlier study. Two-session SRS for large BM in selected patients provided substantial neurological palliation with a low incidence of acute toxic effects. Long-term follow-up showed that two-session SRS achieved durable tumor control rates as well as acceptable treatment-related morbidity. Although only limited conclusions can be drawn from our study results, this treatment method merits being offered to patients with large BM who are in poor condition or are otherwise ineligible for standard care.

## Abbreviations

BM: Brain metastases; SRS: Stereotactic radiosurgery; KPS: Karnofsky performance status; OS: Overall survival; ND: Neurological death; SRT: Stereotactic radiotherapy; RPA: Recursive partitioning analysis; CNS: Central nervous system; WBRT: Whole brain radiotherapy; MR: Magnetic resonance; CT: Computed tomography; LQ: Linear quadratic; TV: Tumor volume; CTCAE: Common terminology criteria for adverse events; RECIST: Response evaluation criteria in solid tumors.

## Competing interests

The authors declare that they have no competing interests.

## Authors’ contributions

SY performed the radiosurgical management of these patients and prepared the manuscript. MH provided critical review of the manuscript for important intellectual content. Both authors read and approved the final manuscript.
